# Comparison of locus-specific databases for *BRCA1* and *BRCA2* variants reveals disparity in variant classification within and among databases

**DOI:** 10.1007/s12687-015-0220-x

**Published:** 2015-03-18

**Authors:** Paris J. Vail, Brian Morris, Aric van Kan, Brianna C. Burdett, Kelsey Moyes, Aaron Theisen, Iain D. Kerr, Richard J. Wenstrup, Julie M. Eggington

**Affiliations:** Myriad Genetic Laboratories, Inc., 320 Wakara Way, Salt Lake City, UT 84108 USA

**Keywords:** *BRCA1*, *BRCA2*, Variant classification, Variants of uncertain significance, Locus-specific variant databases

## Abstract

Genetic variants of uncertain clinical significance (VUSs) are a common outcome of clinical genetic testing. Locus-specific variant databases (LSDBs) have been established for numerous disease-associated genes as a research tool for the interpretation of genetic sequence variants to facilitate variant interpretation via aggregated data. If LSDBs are to be used for clinical practice, consistent and transparent criteria regarding the deposition and interpretation of variants are vital, as variant classifications are often used to make important and irreversible clinical decisions. In this study, we performed a retrospective analysis of 2017 consecutive *BRCA1* and *BRCA2* genetic variants identified from 24,650 consecutive patient samples referred to our laboratory to establish an unbiased dataset representative of the types of variants seen in the US patient population, submitted by clinicians and researchers for *BRCA1* and *BRCA2* testing. We compared the clinical classifications of these variants among five publicly accessible *BRCA1* and *BRCA2* variant databases: BIC, ClinVar, HGMD (paid version), LOVD, and the UMD databases. Our results show substantial disparity of variant classifications among publicly accessible databases. Furthermore, it appears that discrepant classifications are not the result of a single outlier but widespread disagreement among databases. This study also shows that databases sometimes favor a clinical classification when current best practice guidelines (ACMG/AMP/CAP) would suggest an uncertain classification. Although LSDBs have been well established for research applications, our results suggest several challenges preclude their wider use in clinical practice.

**Electronic supplementary material:**

The online version of this article (doi:10.1007/s12687-015-0220-x) contains supplementary material, which is available to authorized users.

## Background

The aim of genetic testing is to determine the etiology of a suspected genetic disorder to influence medical management or provide a diagnosis that can guide genetic counseling. For this reason, genetic variants must be classified accurately and consistently. Professional societies and governmental regulatory agencies have provided general guidance regarding the classification, reporting, and long-term follow-up of variants. Guidelines for the classification of variants were provided in 2007 by the American College of Medical Genetics and Genomics (ACMG) (Richards et al. 2008). The ACMG, in collaboration with the Association for Molecular Pathology (AMP) and the College of American Pathologists (CAP), is currently updating their guidelines towards a more evidence-based system that provides semi-quantitative guidelines for variant classification. In publicly circulated near-final drafts of these guidelines (Lyon et al. 2013; Richards et al. 2014), the ACMG/AMP/CAP will be recommending a multi-tier classification system, grouping variants based upon perceived risk of disease association: pathogenic, likely pathogenic, benign, likely benign, and variant of uncertain significance. These guidelines will recommend that the reporting of novel sequence variants from a clinical diagnostic laboratory must include a clinical interpretation based on the best data available at the time of testing. In clinical practice, laboratories with additional gene-specific information and expertise can use these guidelines to complement their findings. For example, our laboratory has previously outlined its approach to variant interpretation for the *BRCA1* and *BRCA2* genes (Eggington et al. 2014).

Locus-specific variant databases (LSDBs) have evolved as a resource for both researchers and clinicians to interpret the clinical relevance of genetic sequence variants associated with Mendelian disorders. The aim of LSDBs is to facilitate variant interpretation through the use of aggregated data, with variant specific data and classifications provided by researchers, laboratories, and clinicians. The new ACMG/AMP/CAP sequence variant classification guidelines will likely continue to recommend the cautious use of LSDBs to establish whether a genetic variant has been reported as associated with disease (Richards et al. 2008). Recently, a group of researchers and clinicians evaluated and curated the largest public Lynch syndrome (*MLH1*, *MSH2*, *MSH6*, and *PMS2*) variant database and demonstrated that the majority of user-submitted clinical classifications were incorrectly classified within the database when evaluated by evidence-based standards (Thompson et al. 2014). This research demonstrates that a cautious use of LSDBs warrants a thorough review of each database’s evidence-based methodologies for classification. In addition, there are many LSDBs available for clinical variant classification and it is generally well accepted that differences between databases exist (Celli et al. 2012; Mitropoulou et al. 2010). However, there has been no comprehensive review to systematically determine the extent of these discrepancies and, therefore, the potential impact on clinical classification.

In this study, we undertook a cross-comparison of LSDBs to measure concordance across and within five publicly accessible *BRCA1* and *BRCA2* variant databases. Our aim was to analyze whether LSDBs provide a consistent classification for the possible disease association of genetic variants. Our results show substantial disparity of variant classifications among and within publicly accessible variant databases. We also show that the LSDBs in this study failed to provide sufficient evidence to verify the databases’ pathogenic classifications for less straightforward variant classifications, with the exception of genetic variants that have been thoroughly characterized in the literature.

## Methods

**Fig. 1:**
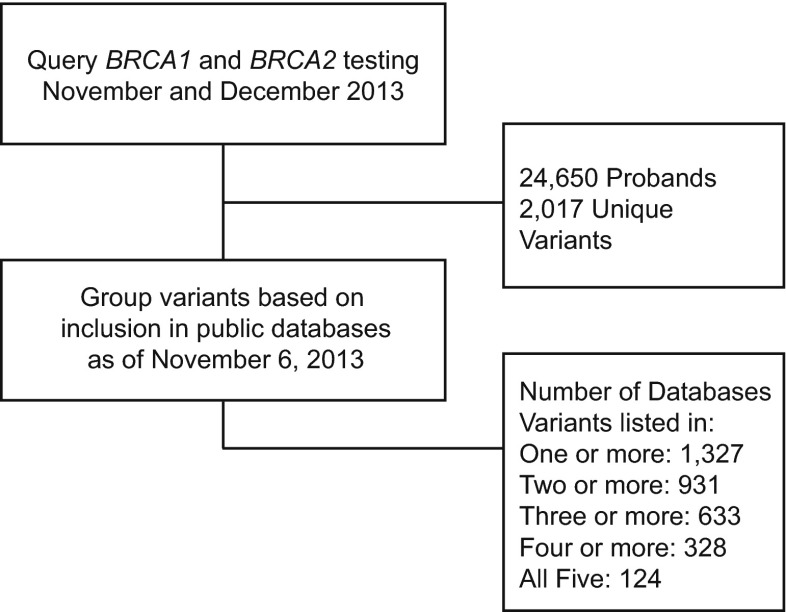
Flow diagram of the study design showing *BRCA1* and *BRCA2* variants identified for study due to detection in the sequencing of 24,650 consecutive patient samples presumed to be unrelated or only distantly related. The number of unique variants among these patients and the number of these variants shared across the BIC, ClinVar, HGMD (paid version), LOVD, and UMD databases are represented

To establish an unbiased dataset for comparison of clinical classifications within and between public databases, we performed a retrospective database query of *BRCA1* and *BRCA2* variants identified among consecutive patients who were referred to Myriad Genetic Laboratories, Inc., for sequencing and large rearrangement testing in November and December of 2013 (Fig. 1). Using this snapshot of data as of November 6, 2013, we then cross-referenced each variant in the dataset with its classification in five publicly accessible databases: (1) the Breast Cancer Information Core (BIC), an online, open-access breast cancer mutation database maintained by the National Human Genome Research Institute at the National Institutes of Health (NIH) (Szabo et al. 2000); (2) the Leiden Open Variation Database 2.0 (LOVD, chromium.liacs.nl/LOVD2/cancer/home.php?select_db=BRCA1, chromium.liacs.nl/LOVD2/cancer/home.php?select_db=BRCA2), maintained by the Leiden University Medical Center, the Netherlands (Fokkema et al. 2011); (3) ClinVar, a freely accessible public archive maintained by The National Center for Biotechnology Information (NCBI) at the NIH with the goal of reporting relationships between human variations and phenotypes (Landrum et al. 2014); (4) the *BRCA1* and *BRCA2* Universal Mutation Database (UMD, http://www.umd.be/BRCA2/), which contains published and unpublished information about *BRCA1* and *BRCA2* mutations reported in a network of 16 French diagnostic laboratories (Beroud et al. 2000); and (5) the Human Gene Mutation Database (HGMD), a paid subscription database maintained by the Institute of Medical Genetics in Cardiff (Stenson et al. 2009).

Variants and their respective classifications in LSDBs were compiled to analyze discrepancy rates between and within databases. To facilitate comparison between different classification schemes, we grouped classifications within databases into three major categories: pathogenic (pathogenic and likely pathogenic), benign (benign and likely benign), and variants of uncertain clinical significance (VUS). The criteria used for group classifications from each database are listed in Table 1. Multiple instances of the same variant, within the same database, were considered as “conflicting” if they were assigned both a pathogenic and benign classification. Classifications were not considered conflicting within the same database if a variant was classified as pathogenic and VUS or benign and VUS. In these cases, the pathogenic or benign clinical classification was used in cross-database comparisons. The variants with classifications that were found in the “Other Classifications” category were excluded from the comparison.

**Table 1:** Definitions of clinical classifications for comparison across databases

	BIC	ClinVar	HGMD	LOVD	UMD
Pathogenic	“Clinically important—yes”	“Pathogenic” “Probably pathogenic”	DM DM? FTV	“+/?” “+?/?”	“5—Causal” “4—Likely causal”
Uncertain (VUS)	“Clinically important—unknown”	“Variant of unknown significance” “Risk factor”	N/A	“?/?”	“3—UV”
Benign	“Clinically important—no”	“No known pathogenicity” “Probably not pathogenic”	FP DP DFP	“−/?” “−?/?”	“1—Neutral” “2—Likely neutral” “Polymorphism”
Other classifications	None	“Not provided” “Conflicting data from submitters”	None	None	“−”

Classifications for HGMD are defined as follows: “DM” is a disease causing (pathological) mutation, “DM?” is a likely disease causing (likely pathological) mutation, “FTV” is a frameshift or truncating variant with no disease association reported yet, “FP” is a polymorphism affecting the structure, function or expression of a gene but with no disease association reported yet, “DP” is a disease associated polymorphism, “DFP” is a disease associated polymorphism with additional supporting functional evidence (Stenson et al. 2009). Classifications for LOVD are defined as follows: “+” is pathogenic, “+?” is probably pathogenic, “?” is effect unknown, “-” is no known pathogenicity, and “-?” is probably no pathogenicity. All classifications are listed in the format “Reported/Concluded”, although for all variants in this data set, the Concluded classification was “?” (Fokkema et al. 2011). A VUS classification in UMD is defined as “3—UV”, referring to “uncertain variant” (Beroud et al. 2000).

For quality assurance of the data, once the classifications in the data set were recorded, a blinded review was performed by two independent reviewers who were given a list of 100 variants from the overall dataset. These 100 variants were randomly selected in order to obtain a representative subset of the variants analyzed here. Each reviewer queried all five databases, noting the classification if available. Once the review was complete, the subset of variants was compared to the initial list of classifications to verify their accuracy. This approach allowed for the discovery and correction of any systematic errors, which arose primarily from inconsistent nomenclature across databases.

### Agreement across databases

**Fig. 2:**
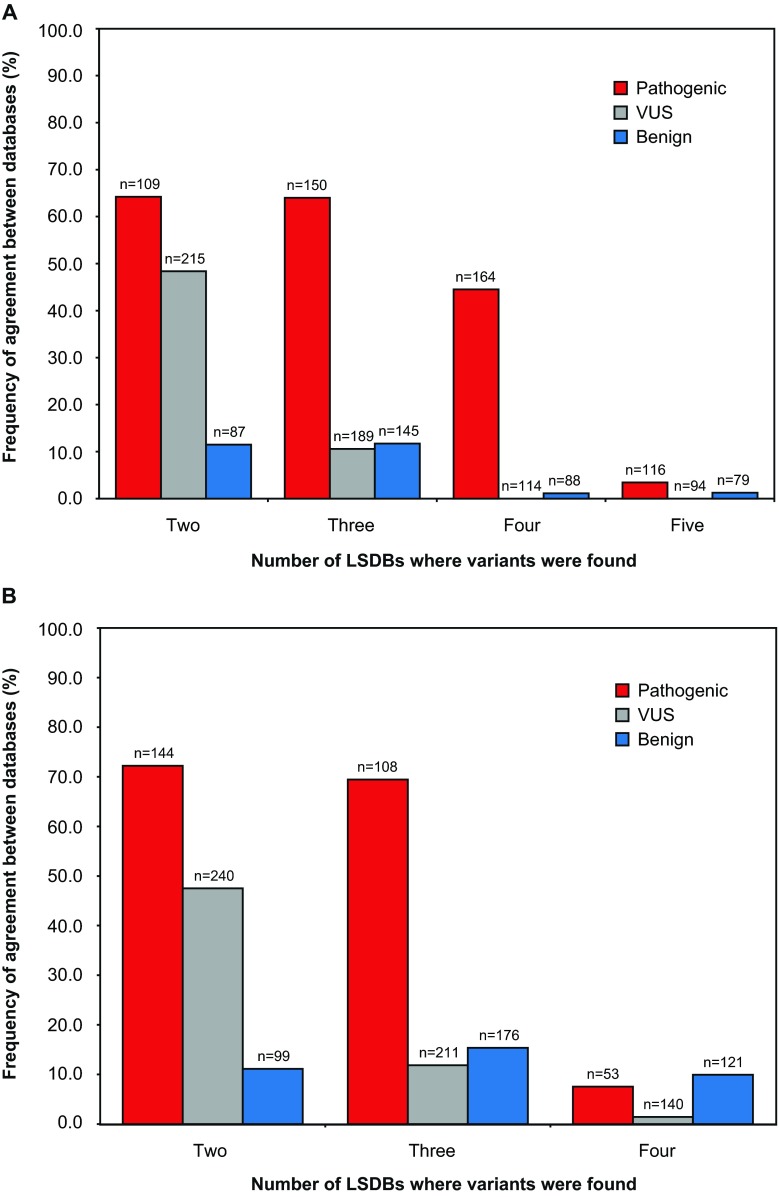
Agreement between database classifications of variants from an unbiased set of 2017 *BRCA1* and *BRCA2* unique genetic variants identified in patients. The proportion of agreement is presented according to the number of databases in agreement (*x*-axis) for (**a**) all databases examined (BIC, ClinVar, HGMD, LOVD, and UMD) and (**b**) all databases except HGMD, which had the highest degree of discrepancy. Variants listed in all databases as pathogenic and likely pathogenic were grouped into the “pathogenic” subset (*red*). Variants listed in all databases as benign or likely benign were grouped into the “benign” subset (*blue*). “VUS” (*grey*) represents variants for which all databases described assigned a classification of variants of uncertain significance

To investigate the degree to which LSDBs agree on variant classifications, we stratified the 2017 unique variants based on the number of databases in which they were present (e.g., all variants that were seen in four databases formed one group). For each database in which a variant occurred, its classification was noted. Agreement was defined as all LSDBs in which a variant occurred assigning the same classification. The frequency of agreement (%) was calculated as the number of variants where agreement was found against the number of variants assigned the specified classification in one or more databases (for sample sizes in the different categories, see Fig. 2). For example, a variant was judged to be in the “pathogenic” subset if at least one database assigned that classification. We recorded this agreement separately for all variants judged to be pathogenic, benign, and VUS.

### Discrepancies within databases

We also examined the potential for conflicting variant classifications to occur within the same database. BIC, HGMD, and UMD provide a single “master” clinical classification per unique variant on the primary variant report page; however, ClinVar and LOVD currently do not provide a single “master” classification and instead list the conflicting entries. The variants with conflicting entries in these databases are listed in the Supplemental Material. We totaled the number of variants seen for each classification (pathogenic, benign, VUS) in each of the five LSDBs and recorded the conflicting classifications when multiple instances of the exact same variant were observed in the same database.

### Analysis of additional evidence utilized by databases

**Fig. 3:**
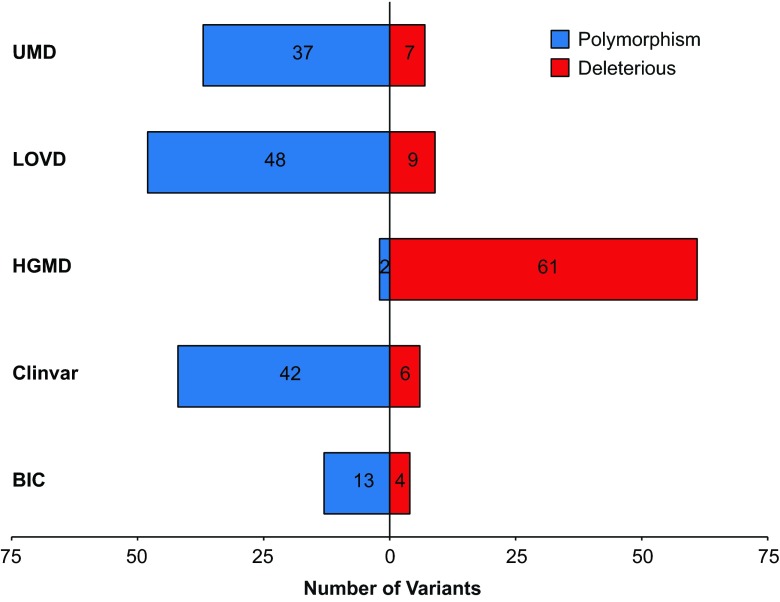
Analysis of the LSDB clinical classifications (benign or pathogenic) of variants considered “VUS” by the criteria outlined in the “Methods” section. Sixty-three of the 124 unique variants shared across the BIC, ClinVar, HGMD (paid version), LOVD, and UMD databases met these criteria. To determine how LSDBs vary in their treatment of these “challenging to classify” variants, we compared the clinical classifications of each “VUS” in all five databases. Variants with conflicting entries of VUS and either pathogenic or benign in the same database are categorized as either pathogenic or benign in the figure (see Table 1). Variants with conflicting classifications of both pathogenic and benign in the same database were excluded from this figure

The use of literature and unique empirical data is an important facet of variant classification. However, the clinical utility of such information may be subject to debate when, e.g., the raw data are unavailable to support a reported conclusion or an intermediate functional effect is reported in a biochemical assay. To assess the use of literature and unique empirical data by LSDBs, we first collected a subset of variants from the 124 unique variants found in all five LSDBs that could be conservatively classified as VUS. The following criteria were used to select this subset: (1) variant listed in all five of the databases, (2) variant receives a default classification of VUS when excluding literature and other empirical evidence, and (3) definitively classified by at least one of the five databases as pathogenic (or likely pathogenic). The draft form of updated guidelines from the ACMG/AMP/CAP Interpretation of Sequence Variations Workgroup was used for this analysis (Table 3). To establish a default VUS classification for criteria #2, the following types of variants were considered: missense variants, intronic variants greater than two nucleotides inside the intron from the native RNA splice acceptor or donor site, in-frame insertions/deletions, and variants within the 5' untranslated region (UTR). We next noted the reported classification of these variants in the five LSDBs and plotted all pathogenic and benign classifications (Fig. 3). Any variants that already held a VUS clinical classification in a given database were excluded from further analysis.

We also evaluated the content of the BIC, ClinVar, LOVD and UMD databases to determine whether they provided sufficient, verifiable supporting data for an independent reviewer to concur with the databases’ pathogenic classification. As HGMD had the highest discrepancy rate when all databases were compared, it was excluded from this analysis. To perform this analysis, we chose a set of “challenging to classify” variants, which we defined as variants present in all four databases (BIC, ClinVar, LOVD, and UMD) that would be conservatively classified as VUS per the criteria listed previously but that were classified as “pathogenic” in one or more databases. The evidence listed in the database was then evaluated using the evidence-based criteria released in draft form by the ACMG/AMP/CAP Interpretation of Sequence Variations Workgroup, which are soon to be published (Lyon et al. 2013; Richards et al. 2014). Sufficient supporting data was defined as verifiable data contained or referenced in the database that met the minimal requirements for a “Likely Pathogenic” classification (note—for the purposes of this evaluation, a variant’s listing in a public database was not used as a supporting line of evidence for classification).

## Results

During the 2-month study period (Nov–Dec 2013), we evaluated the variants identified in 24,650 consecutive patient samples referred to our laboratory for sequencing and large rearrangement testing in the *BRCA1* and *BRCA2* genes. From these patient samples, we obtained 2017 unique *BRCA1* and *BRCA2* variants among the study population. We then categorized these variants by the number of databases in which they were identified. Of the 2017 unique finds, 1327 were present in one or more databases, 931 were present in two or more databases, 633 were present in three or more databases, 328 were present in four or more databases, and 124 were present in all five databases (Fig. 1).

### Agreement across databases

The ability of multiple LSDBs to agree on a given variant classification is illustrated in Fig. 2a. For pathogenic and VUS classifications, it is clear that the level of agreement consistently decreases as more database evaluations are added. For VUSs in particular, there is no agreement once the variant is observed in a least four of the five databases in this study. Although the percentage agreement increases slightly when a “benign” variant is observed in three databases, it falls off again when observed in four. Figure 2b illustrates the agreement analysis, excluding HGMD which had the highest rate of discrepancy, and shows the same general trend as Fig. 2a.

### Discrepancies within databases

Of the 2017 variants evaluated in this study, ClinVar contained 455 variants with 18 of those having conflicting classifications (Table 2). LOVD contained 470 variants with 61 assigned conflicting classifications. These results indicate that when differing classifications are allowed within a database, as is the case for ClinVar and LOVD, discrepancies in clinical classifications may occur.

**Table 2:** Discrepancies within *BRCA1/BRCA2* databases for the 1327 variants evaluated

	BIC	ClinVar	HGMD	LOVD	UMD
A “master” classification is assigned	Yes	No	Yes	No	Yes
Pathogenic classifications	305	172	536	62	201
Benign classifications	84	172	12	256	199
VUS classifications	645	93	0	91	434
Conflicting classifications	N/A	18	N/A	61	N/A
Total unique variants	1034	455	548	470	834

### Analysis of additional evidence utilized by databases

Of the 124 variants shared across all five LSDBs, 63 variants would be conservatively classified as VUS according to the simplified guidelines outlined in the “Methods” section. However, Fig. 3 illustrates that different databases vary significantly in the reported clinical classification for these variants when additional evidence is considered (i.e., literature and unique empirical data). Our results show that HGMD favors pathogenic clinical classifications for 61 of 63 variants in this subset that other databases classify as either VUS or benign (note—HGMD currently states on its Frequently Asked Questions (FAQ) webpage that its current database is contaminated with variants listed as pathogenic that are now known to be benign and that “to resolve this issue is likely to be a slow iterative process.”).

Within the subset of 63 variants identified, 18 were found that could be considered “challenging to classify” (Table 3). Among these 18 variants, without exception, those with sufficient empirical data to support the LSDB pathogenic classifications were those that had been published in peer-reviewed literature. In this subset, UMD and LOVD performed the best in supporting their pathogenic classifications through the use of appropriate literature references (71 % and 40 % of the pathogenic claims, respectively) (Table 3). BIC and ClinVar performed equally poorly, both failing to support any of the pathogenic classifications with empirical evidence at the time of this study.

**Table 3:** Evaluation of the supporting data provided in *BRCA1/2* databases for the pathogenic classification of 18 challenging variants as measured by evidence-based guidelines for variant interpretation of “likely pathogenic”(Lyon et al. 2013; Richards et al. 2014)

	BIC	ClinVar	LOVD	UMD
*BRCA1* c.2351C>T p.Ser784Leu	VUS	Benign Data: none^a^ Insufficient	Pathogenic Data: literature Insufficient	VUS
*BRCA1* c.3082C>T p.Arg1028Cys	VUS	Benign Data: none^a^ Insufficient	Pathogenic Data: literature Insufficient	VUS
*BRCA1* c.4484G>T p.Arg1495Met	Pathogenic Data: none Insufficient	Pathogenic Data: none^a^ Insufficient^b^	Pathogenic Data: literature^c^ Sufficient	Pathogenic Data: literature^d^ Sufficient
*BRCA1* c.4868C>G p.Ala1623Gly	VUS	Pathogenic Data: none^a^ Insufficient^e^	Pathogenic Data: literature Sufficient	Pathogenic Data: literature^d^ Sufficient
*BRCA1* c.5072C>T p.Thr1691Ile	VUS	VUS	Pathogenic Data: literature Insufficient	VUS
*BRCA1* c.4986+6T>C (IVS16+6T>C)	Pathogenic Data: none Insufficient	Pathogenic Data: none^a^ Insufficient^e^	VUS	Pathogenic Data: literature^d^ Sufficient
*BRCA1* c.5074G>C p.Asp1692His	Pathogenic Data: none Insufficient	Pathogenic Data: none^a^ Insufficient^e^	VUS	Pathogenic Data: literature^d^ Insufficient
*BRCA1* c.5408G>C p.Gly1803Ala	VUS	VUS	Benign Data: literature Insufficient	Pathogenic^f^ Data: literature^d^ Insufficient^f^
*BRCA2* c.6290C>T p.Thr2097Met	VUS	Pathogenic Data: none^a,g^ Insufficient^e^	VUS	VUS
*BRCA2* c.6322C>T p.Arg2108Cys	VUS	VUS	Pathogenic Data: literature Insufficient	VUS
*BRCA2* c.7007G>A p.Arg2336His	Pathogenic Data: none Insufficient	Pathogenic Data: none^a^ Insufficient^b^	Pathogenic Data: literature Sufficient	Pathogenic Data: literature^d^ Sufficient
*BRCA2* c.7565C>T p.Ser2522Phe	VUS	VUS	Pathogenic Data: Literature Insufficient	VUS
*BRCA2* c.7868A>G p.His2623Arg	VUS	Pathogenic Data: none^a^ Insufficient^b^	Pathogenic Data: literature Insufficient	VUS
*BRCA2* c.7878G>C p.Trp2626Cys	VUS	VUS	Pathogenic Data: literature Sufficient	VUS
*BRCA2* c.8168A>G p.Asp2723Gly	VUS	VUS	Pathogenic Data: literature Sufficient	Pathogenic Data: literature^d^ Sufficient
*BRCA2* c.9104A>C p.Tyr3035Ser	VUS	VUS	Pathogenic Data: literature Insufficient	VUS
*BRCA2* c.9235G>A p.Val3079Ile	VUS	Pathogenic Data: none^ag^ Insufficient^e^	Benign Data: literature Insufficient	VUS
*BRCA2* c.9371A>T p.Asn3124Ile	VUS	Pathogenic Data: none^a^ Insufficient^b^	Pathogenic Data: literature Sufficient	VUS
Share of pathogenic classifications with sufficient evidence	0/4 0 %	0/11 0 %	6/15 40 %	5/7 71 %

“Pathogenic” or “VUS” refers to the classification made in the associated database for the variant according to the groupings defined in Table 1. For variants listed as pathogenic, “data” describes the type of data provided in the associated database to support the classification. “Sufficient” or “insufficient” represents whether or not the supporting data provided or referenced by the database is verifiable and meets the minimal requirements for a “likely pathogenic” classification according to ACMG/AMP/CAP evidence-based criteria (Lyon et al. 2013; Richards et al. 2014)

^a^Classification was submitted to ClinVar from the Sharing Clinical Reports Project (http://sharingclinicalreports.org/), but no supporting evidence provided in the ClinVar display

^b^At the time of the initial ClinVar evaluation (November 6, 2013), ClinVar database did not provide supporting data for the clinical classification. However, at the time of manuscript preparation (March 11, 2014), the database listed an additional entry for the variant, which lacked a clinical classification, but did provide literature references which *did not* provide sufficient verifiable evidence required to meet minimal ACMG standards for at least a likely pathogenic classification

^c^LOVD listed both pathogenic and uncertain classifications (see Table 1) for this variant where the literature associated with the uncertain classification *did* provide sufficient verifiable evidence to meet minimal ACMG standards for a likely pathogenic classification, but the literature associated with the listed pathogenic classification *did not* meet these standards

^d^UMD frequently provides the age of disease onset in anonymized patients who have been reported by UMD’s contributors as carrying the variant. However, for the variants reported here, literature references were the primary data source for variant classification

^e^Same as table footnote “b” for ClinVar, except that the entry added after our initial evaluation listed literature references which *did* meet minimal ACMG standards for a likely pathogenic classification, though the added entry did not provide a clinical classification

^f^The database lists a pathogenic classification, but the supporting data trends for a contrary (benign) classification

^g^ClinVar displays the most recent classification submitted from the Sharing Clinical Reports Project. At the time of the initial ClinVar evaluation (November 6, 2013), ClinVar database displayed a “pathogenic” classification” from the Sharing Clinical Reports Project. At the time of manuscript preparation (March 11, 2014), ClinVar displayed a “benign” or “likely benign” classification from the Sharing Clinical Reports Project

Of special note, in Table 3 are the conflicting classifications for *BRCA1* c.5408G>C (p.Gly1803Ala) between LOVD and UMD. The latter classifies the variant as pathogenic and the former as benign. The two databases provide overlapping literature citations to support their classifications. However, while UMD classifies the variant as pathogenic, the comments listed for the literature cited in the variant information suggest that the variant is trending towards a benign classification. The pathogenic classification is therefore likely to be a data entry error or an instance where incomplete information is provided to support the given classification. Similar differences were discovered in other databases. For example, a LOVD entry for the well-characterized pathogenic mutation *BRCA1* c.181T>G (p.Cys61Gly) is listed as benign (Caligo et al. 2009; Goldgar et al. 2004). Also discovered were likely data entry errors for two of the 18 variants in Table 3, which were submitted to ClinVar through the Sharing Clinical Reports Project (see Table 3 footnotes). Collectively, this highlights the importance of reviewing all available literature and supporting information regarding a variant classification before issuing a clinical report, as database classifications can be discordant and incomplete.

## Discussion

Accurate and consistent data curation is critical to the value derived from LSDBs in categorizing the relationship between genetic variation and disease, particularly for clinical applications. In this study, we performed a retrospective analysis of 2017 *BRCA1* and *BRCA2* variants identified in our laboratory. We compared the clinical classifications of these variants across BIC, ClinVar, HGMD (paid version), LOVD, and UMD to determine whether LSDBs provide a consistent clinical classification.

Our results show substantial disparity of variant classifications among databases. In our evaluation, it was rare for all five databases to agree on variant classification. For example, of 116 variants present in all five databases that received a classification of “pathogenic” in at least one database, only four variants were classified as pathogenic in all five databases (Fig. 2a). Even when the outlier database (HGMD) was removed from the comparison, this did not mitigate the decreased level of agreement we observed as more databases are considered. These results not only highlight an absence of increased accuracy when a variant is observed in multiple databases but also the disparity in classification schemes used across LSDBs. Although two thirds of the variants identified in the patient samples were listed in one or more *BRCA1* and *BRCA2* databases, the remainders were not listed in any LSDB, likely owing to their rarity.

Two of the five evaluated databases do not provide a “master” clinical classification and allow for internal database conflicts (pathogenic and benign) for the same variant. These conflicts limit the overall utility of LSDBS and highlight the inconsistent methodologies used to populate the database.

As information from genetic testing may be used to make important clinical decisions that may involve prophylactic surgery, the ACMG/AMP/CAP guidelines recommend a conservative and evidence-based approach to variant interpretation, with the goal of avoiding speculation. When sufficient data is absent for an evidence-based classification, the guidelines recommend classifying variants as having uncertain clinical significance. However, it is important to note that all public databases included in our analysis contain a disclaimer that the database is to be used for research purposes only. Our findings illustrate that some public databases frequently lean more towards aggressive classification when evidence-based protocols indicate that, in a clinical setting, a VUS classification would be more appropriate for particular variants.

For an unbiased subset of 18 variants evaluated in this study, application of these evidence-based guidelines to existing US government-sponsored and international-based *BRCA1* and *BRCA2* databases shows that the BIC, ClinVAR, LOVD, and UMD databases failed to provide sufficient verifiable content, independent of published literature, to achieve evidence-based verification of their listed “pathogenic” classifications. This might suggest that the use of a database that relies solely upon literature references would be more prudent, such as the use of HGMD. Yet, as described on the HGMD FAQ web page, this database is known to be significantly contaminated with “pathogenic” variants classified in the literature as benign. This information correlates with our findings that HGMD variant classifications were frequent outliers (Fig. 3). However, not all literature used to support a LSDB’s pathogenic classification provided sufficient evidence to meet the evidence-based standards for “likely pathogenic.” This underscores the need for users to independently evaluate referenced literature. Interestingly, even when published literature provided sufficient evidence for a pathogenic classification, the four databases failed to show concordance in the use of the best available literature.

Recently, Thompson et al. reported an evaluation of the LOVD-hosted InSIGHT database for mismatch repair variants. In this analysis, the application of evidence-based criteria resulted in the reclassification of 66 % of the eligible variant entries, including the reversal of pathogenic classifications to benign and vice versa (Thompson et al. 2014). Considering that most public LSDBs do not include a user-tracking mechanism, it is unlikely that the laboratories, clinicians, and patients who previously used the incorrect, non-evidence-based classifications were actively notified of the variant reclassifications. This poses a significant challenge and burden for the user of public LSDBs that has not yet been resolved.

The ability for laboratories to provide sufficient patient- and family-specific data in public databases is challenging because of country-specific laws protecting personal health information. To counter this, databases that attempt to provide empirical data often simplify complex phenotype/genotype information in efforts to anonymize the data for data sharing. However, such simplified data may be misleading. In *BRCA1* and *BRCA2* LSDBs in which only the proband disease status is provided (e.g., UMD), ascertainment bias must be considered in all assessments as the majority of tested patients are being selected for breast and/or ovarian cancers; thus, truly benign variants may seem to be associated with disease because of this bias. It is therefore critical to develop and use clinically validated methods that rely on rigorous control matching to account for this bias (Eggington et al. 2014; Pruss et al. 2014).

## Conclusions

This analysis presents a systematic evaluation of several commonly used LSDBs and their limitations in clinical variant classification. Our results show substantial disparity of variant classifications among and within publicly accessible variant databases. Although LSDBs have been well established for research applications, our results suggest that several challenges inhibit their wider use in clinical practice. Healthcare providers should exercise caution when using these research tools for clinical purposes. With so many *BRCA1/2*-specific databases now available, it is a challenge to identify the most updated and accurate resources. The number of available databases also presents the challenge of curating the often conflicting variant classifications available (Celli et al. 2012; Mitropoulou et al. 2010). We recommend that for any test that may result in definitive and irreversible clinical intervention, the laboratory issuing the genetic report should carefully evaluate each piece of data used for variant classification.

## Electronic supplementary material

Below is the link to the electronic supplementary material.

ESM 1 (DOCX 32.8 kb)

## References

[CR1] Beroud C, Collod-Beroud G, Boileau C, Soussi T, Junien C (2000). UMD (universal mutation database): a generic software to build and analyze locus-specific databases. Hum Mutat.

[CR2] Caligo MA, Bonatti F, Guidugli L, Aretini P, Galli A (2009). A yeast recombination assay to characterize human BRCA1 missense variants of unknown pathological significance. Hum Mutat.

[CR3] Celli J, Dalgleish R, Vihinen M, Taschner PE, den Dunnen JT (2012). Curating gene variant databases (LSDBs): toward a universal standard. Hum Mutat.

[CR4] Eggington JM (2014). A comprehensive laboratory-based program for classification of variants of uncertain significance in hereditary cancer genes. Clin Genet.

[CR5] Fokkema IF, Taschner PE, Schaafsma GC, Celli J, Laros JF, den Dunnen JT (2011). LOVD v. 2.0: the next generation in gene variant databases. Hum Mutat.

[CR6] Goldgar DE, Easton DF, Deffenbaugh AM, Monteiro AN, Tavtigian SV, Couch FJ, Breast Cancer Information Core Steering C (2004). Integrated evaluation of DNA sequence variants of unknown clinical significance: application to BRCA1 and BRCA2. Am J Hum Genet.

[CR7] Landrum MJ, Lee JM, Riley GR, Jang W, Rubinstein WS, Church DM, Maglott DR (2014). ClinVar: public archive of relationships among sequence variation and human phenotype. Nucleic Acids Res.

[CR8] Lyon E, Richards CS, Hegde MR, Bale SJ, Rehm HL, Gastier-Foster JM (2013) Interpretation of Sequence Variants (ACMG/CAP/AMP). In: Association for Molecular Pathology Annual Meeting, Phoenix, AZ

[CR9] Mitropoulou C, Webb AJ, Mitropoulos K, Brookes AJ, Patrinos GP (2010). Locus-specific database domain and data content analysis: evolution and content maturation toward clinical use. Hum Mutat.

[CR10] Pruss D (2014). Development and validation of a new algorithm for the reclassification of genetic variants identified in the BRCA1 and BRCA2 genes. Breast Cancer Res Treat.

[CR11] Richards CS (2008). ACMG recommendations for standards for interpretation and reporting of sequence variations: revisions 2007. Genet Med : Off J Am Coll Med Genet.

[CR12] Richards CS et al (2014) Labs are from Venus and Docs are from Mars: Interpretation and Reporting of Sequence Variants. In: American College of Medical Genetics and Genomics Annual Clinical Genetics Meeting, Nashville, TN

[CR13] Stenson PD, Mort M, Ball EV, Howells K, Phillips AD, Thomas NS, Cooper DN (2009). The Human Gene Mutation Database: 2008 update. Genome Med.

[CR14] Szabo C, Masiello A, Ryan JF, Brody LC (2000). The breast cancer information core: database design, structure, and scope. Hum Mutat.

[CR15] Thompson BA (2014). Application of a 5-tiered scheme for standardized classification of 2,360 unique mismatch repair gene variants in the InSiGHT locus-specific database. Nat Genet.

