# Supplementary material for: Comparison of locus-specific databases for BRCA1 and BRCA2 variants reveals disparity in variant classification within and among databases
Source: J Community Genet. 2015 Mar 18;6(4):351–9. doi: 10.1007/s12687-015-0220-x (PMC4567983; doi:10.1007/s12687-015-0220-x)
Supplement: Supplementary file 1 — (DOCX 32.8 kb) [file 12687_2015_220_MOESM1_ESM.docx]

**Supplemental Material**

**Vail et al, Comparison of locus-specific databases for *BRCA1* and *BRCA2* variants reveals disparity in variant classification within and among databases**

***Discrepancies within databases***

The conflicting classifications identified in LOVD (Table 1) and ClinVar (Table 2) represent the classification status at the time of manuscript preparation. Database classifications are revised as new information becomes available, database curation occurs, or new versions of a database are developed. As such, several conflicts have been resolved since the initial investigation. It should be noted that no additional analysis was performed to identify new conflicting classifications.

All changes in LOVD classifications represent differences between the new LOVD 3.0 database, which was not yet fully operational at the time of this study. These differences between different versions of the same database are consistent with previous literature findings (Mitropoulou et al. 2010). Additionally, not all databases display the date a classification was updated. Therefore, while some conflicting classifications may be resolved, there is no systematic approach to identify classification updates in order to employ the most recent information in the clinical setting.

**Table 1. Variants with Conflicting* Classifications in LOVD**

| **Gene** | **HGVS Name** | **LOVD Initial Classification** | **Resolved?** | **Current Classification** |
| --- | --- | --- | --- | --- |
| BRCA1 | c.211A>G (p.Arg71Gly) | Conflicting | Yes  (LOVD 3.0) | Pathogenic (+/+) |
| BRCA1 | c.5324T>G (p.Met1775Arg) | Conflicting | No | Conflicting |
| BRCA1 | c.2521C>T (p.Arg841Trp) | Conflicting | Yes  (LOVD 3.0) | Benign (-/-) |
| BRCA1 | c.5297T>G (p.Ile1766Ser) | Conflicting | No | Conflicting |
| BRCA1 | c.5096G>A (p.Arg1699Gln) | Conflicting | Yes  (LOVD 3.0) | Pathogenic (+/+)/Uncertain (?/?) |
| BRCA1 | c.5207T>C (p.Val1736Ala) | Conflicting | Yes  (LOVD 3.0) | Uncertain (?/?) |
| BRCA1 | c.2077G>A (p.Asp693Asn) | Conflicting | Yes  (LOVD 3.0) | Benign (-/-) |
| BRCA1 | c.3113A>G (p.Glu1038Gly) | Conflicting | Yes  (LOVD 3.0) | Benign (-/-) |
| BRCA1 | c.3548A>G (p.Lys1183Arg) | Conflicting | Yes  (LOVD 3.0) | Benign (-/-) |
| BRCA1 | c.4956G>A (p.Met1652Ile) | Conflicting | Yes  (LOVD 3.0) | Benign (-/-) |
| BRCA1 | c.4955T>C (p.Met1652Thr) | Conflicting | Yes  (LOVD 3.0) | Uncertain (?/?) |
| BRCA1 | c.5348T>C (p.Met1783Thr) | Conflicting | Yes  (LOVD 3.0) | Uncertain (?/?) |
| BRCA1 | c.1648A>C (p.Asn550His) | Conflicting | Yes  (LOVD 3.0) | Benign (-/-)/Uncertain (?/?) |
| BRCA1 | c.3713C>T (p.Pro1238Leu) | Conflicting | Yes  (LOVD 3.0) | Benign (-/-)/Uncertain (?/?) |
| BRCA1 | c.2612C>T (p.Pro871Leu) | Conflicting | Yes  (LOVD 3.0) | Benign (-/-) |
| BRCA1 | c.1067A>G (p.Gln356Arg) | Conflicting | Yes  (LOVD 3.0) | Benign (-/-) |
| BRCA1 | c.2596C>T (p.Arg866Cys) | Conflicting | Yes  (LOVD 3.0) | Benign (-/-)/Uncertain (?/?) |
| BRCA1 | c.4837A>G (p.Ser1613Gly) | Conflicting | Yes  (LOVD 3.0) | Benign (-/-) |
| BRCA1 | c.536A>G (p.Tyr179Cys) | Conflicting | Yes  (LOVD 3.0) | Benign (-/-) |
| BRCA1 | c.4991T>C (p.Leu1664Pro) | Conflicting | No | Conflicting |
| BRCA1 | c.181T>G (p.Cys61Gly) | Conflicting | Yes  (LOVD 3.0) | Pathogenic (+/+) |
| BRCA1 | c.736T>G (p.Leu246Val) | Conflicting | Yes  (LOVD 3.0) | Benign (-/-)/Uncertain (?/?) |
| BRCA1 | c.4039A>G (p.Arg1347Gly) | Conflicting | Yes  (LOVD 3.0) | Benign (-/-) |
| BRCA1 | c.2315T>C (p.Val772Ala) | Conflicting | Yes  (LOVD 3.0) | Benign (-/-) |
| BRCA1 | c.1789G>A (p.Glu597Lys) | Conflicting | No | Conflicting |
| BRCA1 | c.2669G>T (p.Gly890Val) | Conflicting | Yes  (LOVD 3.0) | Benign (-/-) |
| BRCA1 | c.5123C>A (p.Ala1708Glu) | Conflicting | Yes  (LOVD 3.0) | Pathogenic (+/+)/Uncertain (?/?) |
| BRCA1 | c.5095C>T (p.Arg1699Trp) | Conflicting | Yes  (LOVD 3.0) | Pathogenic (+/+)/Uncertain (?/?) |
| BRCA1 | c.1137T>G (p.Ile379Met) | Conflicting | Yes  (LOVD 3.0) | Uncertain (?/?) |
| BRCA1 | c.2428A>T (p.Asn810Tyr) | Conflicting | Yes  (LOVD 3.0) | Benign (-/-)/Uncertain (?/?) |
| BRCA1 | c.5252G>A (p.Arg1751Gln) | Conflicting | Yes  (LOVD 3.0) | Benign (-/-) |
| BRCA1 | c.5411T>A (p.Val1804Asp) | Conflicting | Yes  (LOVD 3.0) | Benign (-/-) |
| BRCA1 | c.2419G>T (p.Ala807Ser) | Conflicting | No | Conflicting |
| BRCA1 | c.5359T>A (p.Cys1787Ser) | Conflicting | No | Conflicting |
| BRCA1 | c.5333A>G (p.Asp1778Gly) | Conflicting | No | Conflicting |
| BRCA1 | c.5117G>C (p.Gly1706Ala) | Conflicting | Yes  (LOVD 3.0) | Benign (-/-) |
| BRCA1 | c.1427A>G (p.His476Arg) | Conflicting | No | Conflicting |
| BRCA1 | c.4910C>T (p.Pro1637Leu) | Conflicting | Yes  (LOVD 3.0) | Uncertain (?/?) |
| BRCA1 | c.5144G>A (p.Ser1715Asn) | Conflicting | No | Conflicting |
| BRCA1 | c.2222C>T (p.Ser741Phe) | Conflicting | No | Conflicting |
| BRCA1 | c.5317A>T (p.Thr1773Ser) | Conflicting | No | Conflicting |
| BRCA2 | c.4258G>T (p.Asp1420Tyr) | Conflicting | Yes  (LOVD 3.0) | Benign (-/-) |
| BRCA2 | c.8567A>C (p.Glu2856Ala) | Conflicting | Yes  (LOVD 3.0) | Benign (-/-) |
| BRCA2 | c.4585G>A (p.Gly1529Arg) | Conflicting | Yes  (LOVD 3.0) | Benign (-/-)/Uncertain (?/?) |
| BRCA2 | c.865A>C (p.Asn289His) | Conflicting | Yes  (LOVD 3.0) | Benign (-/-) |
| BRCA2 | c.2971A>G (p.Asn991Asp) | Conflicting | Yes  (LOVD 3.0) | Benign (-/-) |
| BRCA2 | c.7544C>T (p.Thr2515Ile) | Conflicting | Yes  (LOVD 3.0) | Benign (-/-) |
| BRCA2 | c.125A>G (p.Tyr42Cys) | Conflicting | Yes  (LOVD 3.0) | Benign (-/-) |
| BRCA2 | c.4146_4148del (p.Glu1382del) | Conflicting | Yes  (LOVD 3.0) | Uncertain (?/?) |
| BRCA2 | c.5634C>G (p.Asn1878Lys) | Conflicting | Yes  (LOVD 3.0) | Pathogenic (+/+)/Uncertain (?/?) |
| BRCA2 | c.8917C>T (p.Arg2973Cys) | Conflicting | Yes  (LOVD 3.0) | Benign (-/-) |
| BRCA2 | c.8972G>A (p.Arg2991His) | Conflicting | No | Conflicting |
| BRCA2 | c.7504C>T (p.Arg2502Cys) | Conflicting | Yes  (LOVD 3.0) | Uncertain (?/?) |
| BRCA2 | c.8350C>T (p.Arg2784Trp) | Conflicting | Yes  (LOVD 3.0) | Uncertain (?/?) |
| BRCA2 | c.8167G>C (p.Asp2723His) | Conflicting | Yes  (LOVD 3.0) | Pathogenic (+/+)/Uncertain (?/?) |
| BRCA2 | c.8905G>A (p.Val2969Met) | Conflicting | Yes  (LOVD 3.0) | Benign (-/-)/Uncertain (?/?) |
| BRCA2 | c.7522G>A (p.Gly2508Ser) | Conflicting | Yes  (LOVD 3.0) | Uncertain (?/?) |
| BRCA2 | c.7469T>C (p.Ile2490Thr) | Conflicting | Yes  (LOVD 3.0) | Benign (-/-)/Uncertain (?/?) |
| BRCA2 | c.502C>A (p.Pro168Thr) | Conflicting | Yes  (LOVD 3.0) | Benign (-/-)/Uncertain (?/?) |
| BRCA2 | c.9154C>T (p.Arg3052Trp) | Conflicting | Yes  (LOVD 3.0) | Pathogenic (+/+)/Uncertain (?/?) |
| BRCA2 | c.9275A>G (p.Tyr3092Cys) | Conflicting | Yes  (LOVD 3.0) | Uncertain (?/?) |

*A conflicting classification was noted when both a benign and pathogenic classification were listed for the same variant.

**Initial classification taken November 6, 2013. Resolved status and current classification taken as of January 22, 2015. All variants that were not resolved were not present in the LOVD 3.0 dataset.

***LOVD 3.0 is making efforts to provide "Concluded" classifications, which were all uncertain in LOVD 2.0. This effort was in beginning stages at the time of this study and most of the variants in this study had no information in LOVD 3.0, thereby making LOVD 2.0 a better resource at that time. LOVD 3.0 also has limited literature references for each variant, whereas LOVD 2.0 has references for nearly all submissions. LOVD 3.0 also generally has fewer submissions per variant than LOVD 2.0, suggesting that not all submissions were ported over.

****No evidence was given for how or when the resolved variants were reclassified.

**Table 2. Variants with Conflicting* Classifications in ClinVar**

| **Gene** | **HGVS Name** | **ClinVar Initial Classification** | **Resolved?** | **Current Classification** | **Last Evaluated** |
| --- | --- | --- | --- | --- | --- |
| BRCA1 | c.1016dup (p.Val340Glyfs*6) | Conflicting data from submitters | Yes | Pathogenic/Likely Pathogenic | 30-Oct-13 |
| BRCA1 | c.68_69del (p.Glu23Valfs*17) | Conflicting data from submitters | No | Conflicting Data from Submitters (Pathogenic/risk factor) | 24-Jul-14 |
| BRCA1 | c.2681_2682del (p.Lys894Thrfs*8) | Conflicting data from submitters | Yes | Pathogenic/Likely Pathogenic | 27-Mar-14 |
| BRCA1 | c.3481_3491del (p.Glu1161Phefs*3) | Conflicting data from submitters | Yes | Pathogenic/Likely Pathogenic | 24-Jul-14 |
| BRCA1 | c.3756_3759del (p.Ser1253Argfs*10) | Conflicting data from submitters | Yes | Pathogenic/Likely Pathogenic | 23-Jan-14 |
| BRCA1 | c.4065_4068del (p.Asn1355Lysfs*10) | Conflicting data from submitters | Yes | Pathogenic/Likely Pathogenic | 11-Jun-14 |
| BRCA2 | c.5722_5723del (p.Leu1908Argfs*2) | Conflicting data from submitters | Yes | Pathogenic/Likely Pathogenic | 11-Jun-14 |
| BRCA2 | c.5946del (p.Ser1982Argfs*22) | Conflicting data from submitters | No | Conflicting Data from Submitters (Pathogenic/risk factor) | 24-Jul-14 |
| BRCA2 | c.6275_6276del (p.Leu2092Profs*7) | Conflicting data from submitters | Yes | Pathogenic/Likely Pathogenic | 24-Jul-14 |
| BRCA2 | c.6591_6592del (p.Glu2198Asnfs*4) | Conflicting data from submitters | Yes | Pathogenic/Likely Pathogenic | 11-Jun-14 |
| BRCA2 | c.8537_8538del (p.Glu2846Glyfs*22) | Conflicting data from submitters | Yes | Pathogenic/Likely Pathogenic | 24-Jul-14 |
| BRCA1 | c.3607C>T (p.Arg1203*) | Conflicting data from submitters | Yes | Pathogenic/Likely Pathogenic | 11-Jun-14 |
| BRCA1 | c.211A>G (p.Arg71Gly) | Conflicting data from submitters | Yes | Pathogenic/Likely Pathogenic | 7-Oct-13 |
| BRCA1 | c.1175_1214del (p.Leu392Glnfs*5) | Conflicting data from submitters | Yes | Pathogenic/Likely Pathogenic | 11-Jun-14 |
| BRCA1 | c.1556del (p.Lys519Argfs*13) | Conflicting data from submitters | Yes | Pathogenic/Likely Pathogenic | 24-Jul-14 |
| BRCA1 | c.5558dup (p.Tyr1853*) | Conflicting data from submitters | Yes | Pathogenic/Likely Pathogenic | 11-Jun-14 |
| BRCA1 | c.5324T>G (p.Met1775Arg) | Conflicting data from submitters | No | Conflicting Data from Submitters (Pathogenic/ Uncertain Significance/ not provided) | 30-Jul-13 |
| BRCA1 | c.2521C>T (p.Arg841Trp) | Conflicting data from submitters | No | Conflicting Data from Submitters (Pathogenic/Benign/ Uncertain Significance/ not provided) | 27-Mar-14 |

*A conflicting classification was noted when the overall clinical significance for the variant was listed as “Conflicting data from submitters” in ClinVar.

**Initial classification taken November 6, 2013. Updated data was reportedly released on November 7, 2013. Resolved status and current classification taken as of January 22, 2015.

***No evidence was given for how the resolved variants were reclassified.

**References**

Mitropoulou C, Webb AJ, Mitropoulos K, Brookes AJ, Patrinos GP (2010) Locus-specific database domain and data content analysis: evolution and content maturation toward clinical use Human mutation 31:1109-1116 doi:10.1002/humu.21332
